# *HSPA1L* rs1061581 polymorphism is associated with the risk of preeclampsia in Han Chinese women

**DOI:** 10.1042/BSR20194307

**Published:** 2020-02-28

**Authors:** Jinbao Zong, Yan Lin, Qingwu Tian, Xin Zhao, Kaiqiu Chu, Bing Jiang, Shengjun Li, Guirong Sun, Shiguo Liu

**Affiliations:** 1 Clinical Laboratory, the Affiliated Hospital of Qingdao University, Qingdao, China; 2 Obstetrics Department, Qingdao Women and Children’s Hospital, Qingdao, China; 3 Clinical Laboratory, Qingdao Women and Children’s Hospital, Qingdao, China; 4 Medical Genetic Department, the Affiliated Hospital of Qingdao University, Qingdao, China; 5 Prenatal Diagnosis Center, the Affiliated Hospital of Qingdao University, Qingdao, China

**Keywords:** heat shock proteins gene, polymerase chain reaction, preeclampsia, single nucleotide polymorphisms

## Abstract

Preeclampsia (PE) is an excessive systemic inflammation response with dysfunction of endothelial. As a stress protein, heat shock protein 70 (HSP70) plays a pivotal role in protecting cells against apoptosis, oxidative damage and genetic damage. In humans, three genes encode members of the HSP70 class: *HSPA1A, HSPA1B* and *HSPA1L*. Our study was to investigate the association between genetic variations of *HSPA1L* and the susceptibility for PE in Chinese Han population. The polymorphisms of rs2227956, rs1043618 and rs1061581 in *HSPA1L* were genotyped by TaqMan allelic discrimination real time polymerase chain reaction (PCR) in 929 PE patients and 1024 healthy pregnant women. Statistic difference of the genotypic and allelic frequencies were found in *HSPA1L* rs1061581 between PE patients and controls (χ2 = 29.863, *P* < 0.001 by genotype; χ2 = 27.298, *P* < 0.001, OR = 1.874, 95%CI 1.476–2.379 by allele) and *HSPA1L* rs1061581 A alleles occurred more frequently in PE patients compared with healthy controls (PE vs. controls 10.28% vs. 5.76%). Furthermore, we divided the PE cases into early-onset/late-onset PE and mild/severe PE subgroups and found statistical differences in genotypic and allelic frequencies of the *HSPA1L* rs1061581 between early-onset PE, late-onset PE, mild PE, severe PE and controls, respectively. Moreover, *HSPA1L* rs1061581 A alleles were more frequent in early-onset PE, late-onset PE, mild PE and severe PE than controls respectively. Therefore, we concluded that *HSPA1L* rs1061581 polymorphism is associated with the risk of PE in Han Chinese women and A alleles may play a role in the susceptibility for PE.

## Introduction

Pre-eclampsia (PE) is a pregnancy-specific syndrome characterized by *de novo* hypertension (systolic blood pressure higher than 140 mmHg and/or diastolic blood pressure higher than 90 mmHg) and/or proteinuria (>300 mg/24 h) that occurs after 20 weeks of gestation in pregnancies, and is always accompanied with multiorgan disorders, which affects the maternal and fetal mortality and morbidity severely [1–3]. As a multifactorial disorder affecting about 5–7% of all women during pregnancy, to date, a number of studies have been carried out to explore the mechanisms of PE, including hereditary variants, inflammation, immunological imbalance and oxidative stress and so on, the specific pathogenesis has not yet been fully explained [4–6].

Although the etiology of PE is not clear, there is increasing evidence showing that inflammation plays an important role in the development of PE [7]. The normal pregnancy is considered to be a general inflammatory response, while PE is considered to be an enhanced inflammatory response [8]. In addition, PE is regarded as a complex inflammation system acting in network, which not only contains the endothelial but also including the inflammatory immune cells, blood clotting and complement system, metabolic and other changes mainly regulated by cytokines and inflammatory factors [9]. Otherwise, some inflammatory cytokines, such as IL-1β, IL-2, IL-6 and IFN-γ, were higher in the serum of PE patients than that of normotensive pregnancies [10–12], suggesting that inflammation may be involved in the pathogenesis of PE strongly. Oxidative stress is defined as an imbalance between reactive oxygen species (ROS) and antioxidant forces in which ROS prevails the antioxidant forces [13,14]. Strong evidence exists that oxidative stress plays a pivotal role in the pathology of PE [15,16].

As a stress protein, HSP70 plays a pivotal role in protecting cells against apoptosis, oxidative damage and genetic damage [17]. Placental ischemia, oxidative stress and maternal systemic inflammatory response, which play important roles in the pathogenesis of PE, have been shown to induce the expression of HSP70 [18]. Moreover, studies indicated that HSP70 is involved in the pathogenesis of hypertension and associated diseases such as coronary heart disease, atherosclerosis, ischaemic stroke, Parkinson’s disease, high-altitude illness and the forth, and there were high levels of HSP70 in serum, plasma and placental tissue of PE patients [17,19,20]. As several polymorphisms in *HSP70* can influence protein expression or functions in some degree, thus may affect ability in stress tolerance and susceptibility to certain diseases [17]. And the gene variants involved in inflammation, oxidative stress, and thrombophilia were related to in the development in PE [21]. In addition, Andrea Fekete et al. found that *HSPA1L* T(2437)C polymorphisms may be associated with the susceptibility for PE [22]. Therefore, we designed this study to examine whether polymorphisms of three tag SNPs (rs2227956, rs1043618 and rs1061581) in *HSPA1L*, which is one of the three genes encoding HSP70 proteins [22], were associated with the risk of PE or not.

## Materials and methods

### Subjects

About 929 PE patients as the case group and 1024 normotensive pregnencies as the control group were enrolled from the Affiliated Hospital of Qingdao University, Linyi People’s Hospital, Binzhou Medical University Hospital, Yantai Yuhuangding Hospital, Yantaishan Hospital, Liaocheng People’s Hospital and Heze People’s Hospital between November 2017 and February 2019. The case group and the control group were age-matched and all came from Chinese Han population. Clinical characteristics such as maternal age, gestational weeks of admission and delivery, pregnancy and delivery history, clinical symptoms, and results of laboratory examinations were collected in a database through the questionnaire filled out by all the recruited staff.

The PE was diagnosed according to the criteria from the Report of the ‘National High Blood Pressure Education Program’ [23]. The recruited controls were normotensive pregnancies without clinical history of PE. In addition, both PE and control pregnant women are excluded from pre-existing hypertension, chronic hypertension, heart disease, gestational diabetes mellitus, renal disease, thyroid function disorder, uterine malformation, placental abruption, multiple pregnancies, *in vitro* fertilization treatment, cancer, or any other systemic disease, including systemic lupus erythematosus (SLE) and rheumatoid arthritis (RA). This research was approved by the ethics committee of the Affiliated Hospital of Qingdao University and all participants in our study signed the written informed consent.

### Genetic studies

We used a Qiagen DNA extraction kit (Qiagen, Hilden, Germany) to extract DNA from the 300 µl peripheral venous blood of the pregnancies and genotype the polymorphisms of rs2227956, rs1043618 and rs1061581 in HSPA1L by the TaqMan allelic discrimination real-time PCR technology with the Taqman probes and primers synthesized by Applied Biosystems of Life Technologies (New York, U.S.A.). For rs2227956, the sequence of forward and reverse primer is 5′-AATGGTATTCTCAATGTCAC AGCCA-3′ and 5′-GGACAAGAGCACCGGCAAGGTGAAC-3′ respectively; for rs1043618, the forward and reverse primer is 5′-CGTTTCCAGCCCCCAATCTCAGAGC-3′ and 5′-GAGCCGACAGAGAGCAGGGAACCGG-3′ respectively; for rs1061581, the forward primer is 5′-TCCCCAAGGTGCAGAAGCTGCTGCA-3′ and the reverse primer is 5′- GACTTCTTCAACGGGCGCGACCTGA-3′. The PCR was conducted in 25 μl reaction mixture, including 1.25 μl 20 × SNP Genotyping Assay, 12.5 μl 2 × PCR Master Mix and 11.25 μl DNA and DNase-free water. The amplifications were carried out by C1000™ thermal cycler and CFX96™ real-time system (Bio-Rad, Hercules, CA) with the following conditions: 95°C for 3 min, then 45 cycles of 95°C for 15 s and 60°C for 1 min. For each cycle, the fluorescent signals from the VIC- or FAM-labeled probes was detected. Bio-Rad CFX manager 3.0software was used to conduct the genotyping.

### Statistical analysis

All analyses were conducted by statistical software package SPSS 21.0 (SPSS Inc., Chicago, IL, U.S.A.). In order to value whether the control group was representative, we use the chi-square test to assess Hardy–Weinberg equilibrium (HWE) in the controls. Student’s *t*-test was used to compare differences between cases and controls in demographic and clinical characteristics. Statistical significance was set at *P* < 0.05 (two-sided). We used Pearson’s χ2 test (if expected values were below 5, Fisher’s exact test was used) to compare differences in allelic and genotypic distributions between two groups and a significance threshold of *P* = 0.016 (*P*_0_/*N, P*_0_ = 0.05, *N* = 3 SNPs) (two sided) was required when a formal Bonferroni’s correction for the number of SNPs was analyzed. The relative risk degree was showed by odds ratios (ORs) and 95% confidence intervals (CIs).

## Result

### Demographic and clinical characteristics

Table 1 showed the comparison in demographic and clinical characteristics between cases and controls. The mean age of cases and controls was 30.33 ± 5.29 and 30.55 ± 4.14 years old respectively, which was matched between both groups. Compared with the healthy pregnant women, the PE patients have earlier gestational weeks at admission (35.31 ± 3.68 weeks vs. 39.02 ± 1.56 weeks, *P* < 0.001), gestational weeks at delivery (36.13 ± 3.26 weeks vs. 39.35 ± 1.29 weeks, *P* < 0.001), lower birth weight of offspring (2579.12 ± 953.79 g vs. 3411.18 ± 366.16 g, *P* < 0.001), higher Systolic blood pressure (P < 0.001) and Diastolic blood pressure (P < 0.001), and higher levels of Urea nitrogen (*P* < 0.001), Creatinine (*P* < 0.001), ALT (*P* < 0.001), and AST (*P* < 0.001). However, no significant differences were found between the cases and controls in time of pregnancies, number of abortions, white blood cell, neutrophil, triglycerides and total cholesterol.

**Table 1:** Clinical characteristics

	Cases	Controls	*t*	*P*-value
Age (years)	30.33 ± 5.29	30.55 ± 4.14	−1.011	0.312
Gestational age at admission (weeks)	35.31 ± 3.68	39.02 ± 1.56	−28.302	*P* < 0.001
Gestational age at delivery (weeks)	36.13 ± 3.26	39.35 ± 1.29	−27.365	*P* < 0.001
Time of pregnancy	2.18 ± 1.23	2.22 ± 1.21	−0.81	0.418
Number of abortion	0.60 ± 0.93	0.68 ± 0.90	−1.908	0.057
Birth weight of offspring (g)	2579.12 ± 953.79	3411.18 ± 366.16	−24.257	*P* < 0.001
Systolic blood pressure (mmHg)	159.39 ± 19.09	114.26 ± 10.02	65.606	*P* < 0.001
Diastolic blood pressure (mmHg)	103.31 ± 13.59	73.78 ± 7.98	58.648	*P* < 0.001
Urea nitrogen (mmol/l)	4.65 ± 2.05	3.45 ± 3.26	9.074	*P* < 0.001
Creatinine (µmol/l)	67.68 ± 27.07	55.55 ± 15.27	11.029	*P* < 0.001
ALT(IU/l)	24.68 ± 45.89	15.03 ± 13.76	5.674	*P* < 0.001
AST(IU/l)	28.77 ± 47.89	19.45 ± 13.29	5.268	*P* < 0.001

### Genotypic and allelic frequencies

Table 2 showed the genotypic and allelic frequencies of the *HSPA1L* rs2227956, rs1043618 and rs1061581 in cases and controls. The participants of control group in our study were in accordance with HWE for the three SNPs suggesting that they had a group representative (rs2227956, χ2 = 3.076, *P* = 0.0794; rs1043618, χ2 = 2.277, *P* = 0.1313; rs1061581, χ2 = 3.828, *P* = 0.0504). For the PE patients and healthy pregnant women, we found a significant difference in genotypic and allelic frequencies of *HSPA1L* rs1061581 polymorphism (χ2 = 29.863, *P* < 0.001 by genotype; χ2 = 27.298, *P* < 0.001, OR = 1.874, 95%CI 1.476–2.379 by allele), and *HSPA1L* rs1061581 A alleles occurred more frequently in PE patients compared with healthy controls (PE vs. controls 10.28% vs. 5.76%). However, no statistical significances were observed for rs2227956 and rs1043618 between the two groups in terms of genotypic frequencies (rs2227956, χ2 = 0.396, *P* = 0.82; rs1043618, χ2 = 0.731, *P* = 0.694), nor for allelic frequencies (rs2227956, χ2 = 0.009, *P* = 0.924, OR = 0.993, 95%CI 0.850–1.159; rs1043618, χ2 = 0.103, *P* = 0.748, OR = 1.022, 95%CI 0.896–1.164).

**Table 2:** The genotypic and allelic frequencies of rs2227956、rs1043618 and rs1061581 in cases and controls

		Rs2227956					Rs1043618					Rs1061581				
Group	N	CC	CT	TT	C	T	CC	CG	GG	C	G	AA	AG	GG	A	G
PE	929	53	`280	596	386	1472	141	394	394	676	1182	0	191	738	191	1667
Control	1024	54	320	650	428	1620	143	449	432	735	1313	0	118	906	118	1930
χ^2^		0.396			0.009		0.731			0.103		29.863			27.298	
*P*-value		0.82			0.924		0.694			0.748		<0.001			<0.001	
OR					0.993					1.022					1.874	
95%CI					0.850–1.159					0.896–1.164					1.476–2.379	

In order to further explore the relationship between the polymorphisms of *HSPA1L* and PE, we divided the PE patients into early-onset PE and late-onset PE according to the gestational age at diagnosis (early-onset PE defined as those diagnosed before the 34th week of gestation, known to be more severely affected than those with late-onset PE [24]). Through analyzing the Table 3, we can find significant differences in genotypic and allelic frequencies of *HSPA1L* rs1061581 polymorphism between early-onset /late-onset PE and controls (early-onset PE vs. controls: χ2 = 26.134, *P* < 0.001 by genotype; χ2 = 24.073, *P* < 0.001, OR = 2.003, 95%CI 1.511–2.655 by allele; late-onset PE vs. controls: χ2 = 14.009, *P* < 0.001 by genotype; χ2 = 12.966, *P* < 0.001, OR = 1.669, 95%CI 1.260–2.211 by allele). *HSPA1L* rs1061581 A alleles occurred more frequently in early-onset PE and late-onset PE than controls respectively (early-onset PE vs. controls:10.91% vs. 5.76%, late-onset PE vs. controls: 9.26% vs. 5.76%). Nevertheless, no statistical differences in genotypic and allelic frequencies were found between early-onset /late -onset PE and controls in rs2227956 and rs1043618.

**Table 3:** The comparison of genotype distributions and allelic frequencies between early/late-onset PE and control groups

		Rs2227956					Rs1043618					Rs1061581				
Group	N	CC	CT	TT	C	T	CC	CG	GG	C	G	AA	AG	GG	A	G
Early-onset PE	440	26	126	288	178	702	68	195	177	331	549	0	96	344	96	784
Control	1024	54	320	650	428	1620	143	449	432	735	1313	0	118	906	118	1930
χ^2^		1.106			0.169		0.774			0.791		26.134			24.073	
*P*-value		0.575			0.681		0.679			0.374		<0.001			<0.001	
OR					0.960					1.077					2.003	
95%CI				0.789-1.168			0.915-1.268			1.511-2.655						
Late-onset PE	513	29	163	321	221	805	76	210	227	362	664	0	95	418	95	931
Control	1024	54	320	650	428	1620	143	449	432	735	1313	0	118	906	118	1930
χ^2^		0.165			0.169		1.188			0.109		14.009			12.966	
*P*-value		0.921			0.681		0.552			0.741		<0.001			<0.001	
OR					1.039					0.974					1.669	
95%CI				0.865−1.248			0.833-1.139			1.260−2.211						

Moreover, based on guidelines from the American College of Obstetricians and Gynecologists, we divided PE patients into mild and severe PE groups so that we can further explore the association between the polymorphisms of *HSPA1L* and PE [25]. Table 4 displays the analyzed results in detail. There were significant differences in genotypic and allelic frequencies of *HSPA1L* rs1061581 polymorphisms between mild/severe PE and controls (mild PE vs. controls: χ2 = 16.200, *P* < 0.001 by genotype; χ2 = 15.046, *P* < 0.001, OR = 1.997, 95%CI 1.400–2.849 by allele; severe PE vs. controls: χ2 = 22.111, *P* < 0.001 by genotype; χ2 = 20.335, *P* < 0.001, OR = 1.786, 95%CI 1.384–2.305 by allele). *HSPA1L* rs1061581 A alleles occurred more frequently in mild PE and severe PE than controls respectively (mild PE vs. controls: 10.88% vs. 5.76%, severe PE vs. controls: 9.85% vs. 5.76%). However, we failed to find significant differences in the genotypic and allelic frequencies of rs2227956 and rs1043618 between mild/severe PE and controls.

**Table 4:** The comparison of genotype distributions and allelic frequencies between mild/severe PE and control groups

		Rs2227956					Rs1043618					Rs1061581				
Group	N	CC	CT	TT	C	T	CC	CG	GG	C	G	AA	AG	GG	A	G
Mild PE	216	14	70	132	98	334	31	82	103	144	288	0	47	169	47	385
Control	1024	54	320	650	428	1620	143	449	432	735	1313	0	118	906	118	1930
χ^2^		0.709	0.682		2.711			1.018		16.200			15.046			
*P*-value		0.702			0.409		0.258			0.313		<0.001			<0.001	
OR					1.111					0.893					1.997	
95%CI				0.866−1.425			0.717−1.112			1.400−2.849						
Severe PE	711	40	209	462	289	1133	110	309	292	529	893	0	140	571	140	1282
Control	1024	54	320	650	428	1620	143	449	432	735	1313	0	118	906	118	1930
χ^2^		0.717			0.169		0.793			0.624		22.111			20.335	
*P*-value		0.699			0.681		0.673			0.429		<0.001			<0.001	
OR					0.965					1.058					1.786	
95%CI				0.817−1.141			0.92−1.218			1.384−2.305						

## Discussion

PE is a common systemic obstetric disorder whose specific pathogenesis has not yet been fully explained. However, it is becoming increasingly accepted that oxidative stress, and maternal systemic inflammatory response have a key role in the pathogenesis of PE [8,15,16], which may induce the expression of HSP70 [18].

Heat shock proteins (HSPs) are highly conserved molecules that range from 10 to 150 kDa in molecular mass and function as major molecular chaperones responding to a variety of stress stimuli and maintaining protein homeostasis [26] and are found in all major cellular compartments. Up to now, three major families of HSPs have been revealed: low molecular mass HSPs (16−47 kDa), HSP70 (68−73 kDa), HSP90 (85−90 kDa) [27]. As one of the major HSPs, HSP70 is essential for a cell’s machinery and involved in varieties processes including protein folding, mediating cytoprotective, antiapoptotic effects and regulating immune response [28]. Recent studies have demonstrated that the expression of HSP70 increased in cerebral, myocardial and renal ischemia [29–31]. Furthermore, HSP70 was related to the pathogenesis of hypertension and associated diseases [17,19] and there were high levels of HSP70 in serum, plasma and placental tissue of PE patients [20].

Molvarec et al. denote that elevated levels of circulating HSP70 in PE patients may indicate oxidative stress, systemic inflammation and hepatocellular injury, which not only might play an important role in the pathogenesis of PE, but also could be a pivotal marker for the pathogenesis of PE [32]. Furthermore, when incubating with HSP70, human monocytes presented a rapid intracellular calcium flux, activated nuclear factor-kB (NF-kB), and up-regulated the expression of proinflammatory cytokines [33]. And several studies demonstrated increased NF-kB activation in pregnancies with PE [34–36]. Therefore, we may conclude that NF-kB signaling is one of pathways that elevated levels of circulating HSP70 participate in the pathogenesis of PE. NF-kB is a hetero- or homo-dimer that consists of five subunits of the Rel family of polypeptides: NF-kB1 (p50/p105), NFkB2 (p52/p100), RelA (p65), c-Rel and RelB. It mainly exists in the form of the heterodimer p65/p50 [37]. Before activation, most NF-kB molecules are located in the cytoplasm bounding to a inhibitor of NF-kB protein (IkB). When stimulated, e.g. by HSP70, inflammatory cytokines or ROS, the IkB kinase (IKK) complex is activated, which allows NF-kB to release from the IkB and to translocate to the nucleus. After arriving in the nucleus, NF-kB will bind to kB-regulatory elements of DNA and coordinate transcription activation of plentiful genes involved in vascular inflammation, such as adhesion molecules, chemokines and cytokines, which are highly associated with endothelial dysfunction and the pathogenesis of PE [37,38].

A previous study indicated that the heat shock transcriptional regulatory factors of genetically hypertensive animals were enhanced in activation which coupled to polymorphisms of *HSP70* [39,40]. In humans, three genes encode members of the HSP70 class: *HSPA1A, HSPA1B* and *HSPA1L* localized on chromosome 6p21.3 [22]. *HSPA1A* and *HSPA1B* encode identical heat shock-inducible protein products, whereas the *HSPA1L* encodes a non-heat inducible protein that shares 90% sequence identity with the heat shock-inducible protein products [41]. Andrea Fekete et al. found that *HSPA1L* (2437)CC genotypes were more frequent among PE patients than controls, suggesting that *HSPA1L* T(2437)C polymorphisms may be related with the susceptibility for PE [22]. Therefore, we chosen three tag SNPs (rs2227956, rs1043618 and rs1061581) in *HSPA1L* gene and conducted genotyping and analyzing to investigate the relationship between polymorphisms of the three SNPs in *HSPA1L* and the susceptibility for PE.

In the present study, we observed significant differences in genotypic and allelic frequencies of rs1061581 between the PE patients and the normotensive pregnancies, which can demonstrate an association between *HSPA1L* rs1061581 polymorphism and the susceptibility for PE. Moreover, *HSPA1L* rs1061581 A alleles occurred more frequently in PE patients compared with healthy controls (PE vs. controls 10.28% vs. 5.76%), suggesting that *HSPA1L* rs1061581 A alleles may play a role in the susceptibility for PE. However, we failed to reveal an association between the polymorphisms of other two SNPs and PE. Furthermore, the polymorphism of *HSPA1L* rs1061581 was found to be associated with early-onset PE, late-onset PE, mild PE and severe PE respectively and *HSPA1L* rs1061581 A alleles were more frequent in early-onset PE, late-onset PE, mild PE and severe PE than controls respectively, indicating that *HSPA1L* rs1061581 A alleles may play a role in the susceptibility for PE further. Whereas, for rs2227956 and rs1043618, there were no significant association between the polymorphisms and early-onset /late-onset /mild /severe PE, neither. Therefore, it can be concluded that the present study provides apparent evidence that polymorphism of *HSPA1L* rs1061581 is associated with the risk of PE in Han Chinese women. However, larger-scale and well-designed studies involving different races and regions with environmental analyses are necessary to be performed to confirm our findings and further investigate the pathogenesis of PE.

## Abbreviations

HSP70: heat shock protein 70
HWE: Hardy–Weinberg equilibrium
NF-kB: nuclear factor-kB
PCR: polymerase chain reaction
PE: preeclampsia
